# Illustrations of the Comparative Anatomy of the Nervous System

**Published:** 1836-07

**Authors:** 


Art. VI.—*Illustrations of the Comparative Anatomy of the Nervous System.* By Joseph Swan.—Part I. 4to. pp. 27. *London*, 1835.

This is one of those works which are much wanted in the English language. The amazing strides which have been made in the knowledge of comparative anatomy by our continental brethren have opened such a field of practical information, that we have been surprised that so few of our own countrymen have directed their attention to this subject. It was the wonder of the great Cuvier, when he visited England, that the treasures in comparative anatomy which this country possesses in the museum of John Hunter, should have been so long unknown, and almost forgotten. Indeed, many of those subjects which had been fully understood and exemplified by dissections by that illustrious anatomist, owing to the comparatively little attention that has been paid to this branch of study in England, have only become known to us through the labours of foreigners. Works, therefore, of the description which Mr. Swan has undertaken, illustrative of the various structures of the body in different classes of the animal kingdom, are not only desirable, but absolutely necessary, in the present state of our knowledge.

The work before us (of which, however, we have only the first part,) is well calculated, to a certain extent, to forward the object it professes to illustrate, viz. a knowledge of the anatomy of the nervous system in different animals. We must, however, in the outset, take the liberty of finding a little fault with the arrangement which Mr. Swan has chosen to adopt; for, although the work will convey to the student a knowledge of individual structures, it can give him but little idea of their *comparative* anatomy, since this depends upon such an arrangement of the subject as will illustrate the gradual and progressive development of structure from the simplest to the highest forms. The author remarks in his preface that, after much consideration, he had selected "such examples as would explain the scheme on which the nervous system is generally founded;" but we are of opinion that this object will not be forwarded by the arrangement he has chosen. Instead of commencing with the higher Crustacea, in which the nervous system, after having passed through many phases, has acquired a concentrated and high form of development, it would have been better if he had begun with its lowest condition, and gradually ascended through the different classes of animals, showing the principal links and connexions in the series. This would have made the work far more useful than it will now be found, although the excellent plates, being as they are from actual dissection, cannot fail to be highly serviceable. We perfectly agree with the author that actual dissections should be encouraged, and it is with this feeling that we would strongly recommend his work as a valuable collection of dissections. There are a few points more important than the arrangement, upon which we must say a word or two.

We have been much disappointed with regard to the quantity of letterpress which accompanies these beautiful plates, there being scarcely more than the most meager description of the objects represented. In the first five plates there is an entire absence of letters for reference, and description of the particular parts of the objects, by which alone they could be recognized. This most decidedly is a fault. It is only the junior student, or the student who has advanced but to a certain extent, who can be materially assisted by these delineations of structure in the invertebrata; and to these it is more particularly necessary that the objects should be distinctly and accurately represented and described.

The most important qualifications of an anatomical writer are precision and accuracy of description, and fidelity of representation. If these be wanting, however beautiful a work may appear, it ultimately becomes comparatively useless; because, when a student begins to discover, as he advances in information, that that on which he had reposed the most implicit reliance, and perhaps had consulted upon every occasion, contains inaccuracies and errors, he is disposed for a time to disregard it entirely; and it is only when he has arrived at a full knowledge of this subject, and is enabled to distinguish its merits from its imperfections, that he will again turn to it with satisfaction and pleasure. We are sorry to be obliged to notice a few inaccuracies of this kind in the present work, both in the plates themselves and in the letter-press which accompanies them. These are confined chiefly to the structures of invertebrata, with which Mr. Swan evidently is not so well acquainted as with those of the higher animals. We were not a little surprised at finding him mistaking the very principles of development upon which the nervous system is founded. He seems to have adopted the opinion of Ackermann, Reil, and some of the older anatomists, that the sympathetic of the vertebrata is the analogue of the whole nervous system of the invertebrata. At first we could scarcely believe that so able and experienced an anatomist as Mr. Swan should have so completely misunderstood the subject; but we give his own words.

"The different appearance of the nerves encircling the œsophagus, and forming a connexion between the brain and first ganglion in invertebrated animals, can sustain only a very slight claim to a comparison with that between the brain and spinal marrow of the vertebrated. If any other be allowable, it is with the par vagum of those animals, in which the sympathetic nerve becomes so combined with the ganglia of the dorsal nerves as not to admit of a separation. These compound ganglia terminate after the usual manner of the dorsal nerves in the muscles and skin, except that some of them send branches to the viscera. If, therefore, the spinal cord were removed, and the ganglia with the prolongations of each side were drawn together, through the connexion of the par vagum with the brain, a nervous chord similar to that of many invertebrated animals would be produced. This would be a different combination; but it might be well suited to the lower degree of the animal. It may not be necessary in these creatures for the nerves of sensation and motion to be separate, but it may suffice to have a chord of a mixed character, and capable of conferring both qualities, as in the glosso-pharyngeal nerve in man, the par vagum, and some others." (P. 19.)

"In the human fœtus, the sympathetic as well as the spinal nerves and their ganglia are formed at an early period, and when very little more is apparent than the membranes of the spinal marrow. This circumstance favours the opinion, that the long chord of invertebrated animals is not the prototype of the spinal marrow, but of the spinal ganglia and their nerves, the sympathetic and par vagum, according to the preceding observations." (P. 20.)

We are the more astonished at the expression of this opinion by Mr. Swan, who has been so long engaged upon these structures, after the complete identity of the nervous chords of articulata, and the brain and ganglia of mollusca, with the cerebro-spinal system of man and the higher animals, has been so fully made out by such authorities as Meckel, Cuvier, Blumenbach, Gall, Spurzheim, Serres, Rudolphi, and Weber, and still more recently by Müller, Treviranus, and others. Even Mr. Swan himself seems half inclined to take a similar view of the subject, notwithstanding his expressed opinion, when he tells us that the cerebral and œsophageal ganglia of the invertebrata can bear only a very slight comparison with the cerebro-spinal system of vertebrata. But to compare the whole nervous system of the lower animals with the par vagum system only of the higher is truly absurd. Had our author taken the trouble to refer to Müller's paper on the Sympathetic System of Insects in the fourteenth vol. of the Physico-Medical Transactions of Bonn, or to the more recently published paper of Mr. Newport on the Sphinx Ligustri, in the Philosophical Transactions for 1834, he would at once have seen that the analogy between the spinal column, vagus, and sympathetic systems of man and the higher animals, and the corresponding parts of the nervous system in invertebrata, is distinctly identified. The existence of distinct motor and sensitive tracts in invertebrata is also questioned, although it is more than once stated that there is a difference in the appearance of the dorsal and ventral surfaces of the chords in the lobster; that "there is a ganglion inserted into the ventral, and a layer of longitudinal fibres passing over the dorsal," and a tolerable representation of these is given in the plates of the lobster. We think that Mr. Swan ought at least to have done justice to those among his countrymen who have preceded him in publication, and to have made some reference to the paper of Mr. Newport above alluded to, in which these parts are particularly described and delineated. Of the correctness of the facts there stated he might have assured himself by examining the original preparation, which is deposited, we believe, in the Museum of the College of Surgeons.

Another fact appears also to have presented much difficulty to Mr. Swan, which a better acquaintance with the structures of invertebrated animals would have entirely removed. It is the insertion of the ganglia into the abdominal chords. These ganglia were long ago conceived by Treviranus and E. H. Weber to be analogous to the intervertebral ganglia of the spinal cord of vertebrata, and this, as Müller has recently remarked,* the observations of Mr. Newport upon the cerebro-spinal chords of Insects and Crustacea have almost proved.

Passing over some minor errors of delineation, there are certain inaccuracies and omissions in the description and representation of the nervous system of the Centipede which we cannot omit to notice. In this animal, besides the brain and first subœsophageal ganglia, which encircle the œsophagus, there are two double cords, united at certain distances by ganglia, which, as in the lobster, are inserted into their ventral surface. Besides these, there is a small band of nervous matter which extends along the median line above the cords, but lying close to them, from the head to the anus, and just above each ganglion it gives off a few filaments which join with the nerves that proceed from the sensitive and motor columns beneath it. This latter cord, which is very easily observed, is not represented in Mr. Swan's delineation of the Centipede, nor is even its existence alluded to in the letter-press. This is the more remarkable since it has not only been noticed by several authors, and has been minutely

* *Archiv. für Anat.* Jahrgang. 1835. No. I. p. 81 et seq.

delineated in the paper just referred to, by Mr. Newport, but has even been noticed by Mr. Swan himself in the second vol. of the Medical Gazette, for September 27, 1834, p. 317; from which it is clear that he is acquainted with the existence and structure of a part possessing peculiar interest to the physiologist.

Having thus noticed its errors, we turn with much satisfaction, to advert to the great merits and beauties of this publication. The sixth and seventh plates, which represent the sympathetic and spinal nerves of the cod-fish (*Gadus morrhua*), are beyond all praise. Here Mr. Swan is completely master of his subject, and he has performed his task with the greatest ability. The sympathetic nerve and its origin are beautifully shown; its course beneath the branches of the fifth, its junction with the gastric branch of the vagus, its anastomoses around the large blood-vessels, its course on each side of the spinal artery, and its distribution to the spermatic vessels, all evince the labours of the most masterly anatomist. The seventh plate exhibits several anatomical details of great importance. In it the fifth pair of nerves, besides being distributed to the head, are shown to communicate with all the organs of locomotion, the jugular, pectoral, dorsal, ventral, abdominal and caudal fins. Before the nerve is distributed to these parts, it is joined by a branch of the vagus, while the vagus sends two other branches immediately beneath the skin along the sides of the body to the tail, one of them passing along the back, and the other along the abdomen. This is a most interesting distribution, but that of the spinal nerves is still more so.

"Each anterior and posterior bundle, issuing from the spinal chord, is divided into two branches. One branch from the anterior bundle is joined by one from the posterior, and passes forward to the muscles and skin on the anterior part of the spine; the other branch of the anterior bundle, after it has communicated with the posterior branch passing forward, passes backward and is joined by the second of the next posterior bundle, to terminate on the muscles and skin on the posterior part of the spine. Neither of the branches of the posterior bundle forms a ganglion. The same disposition of the spinal nerves is continued to within a short distance from the tail, when the whole of each anterior and posterior bundle becomes joined, and the division into branches for the muscles and skin, on the anterior and posterior parts of the spine, takes place. The spinal marrow terminates in a bulb near the tail. The great simplicity of combination of the spinal nerves of the cod proves that the anterior bundles are not for exciting one set of muscles, and the posterior their antagonists, as it is clearly demonstrated that one portion of the anterior supplies the muscles on the anterior part of the body, and the other those on the posterior. As ganglia are not attached to the posterior bundles, it is clear that these bodies are not an indispensable adjunct to the nerves of sensation." (P. 25.)

We have ourselves noticed a nearly similar distribution of the spinal nerves in the great shark (*Squalus maximus.*) In this animal, one of the highest of the cartilaginous fishes, there are indeed some very slight enlargements upon the posterior roots, but these in some instances are so indistinct as to lead us to doubt whether they ought really to be considered as ganglia. We know of no class of vertebrated animals besides fishes in which the ganglia of the posterior roots are wanting, and hence it becomes difficult to understand why in this class alone they are absent. It is well remarked by Mr. Swan that the simplicity of combination in the spinal nerves of the cod proves that the anterior bundles are not for exciting one set of muscles, and the posterior their antagonists, thus negatively confirming the already fully established views of Bell, Magendie, and others, that these two sets are, one for motion and the other for sensation. We cannot quite agree with our author in his views with regard to the functions of the fifth nerve, and its independence of the will of the animal, when influenced by external impressions; nor with some other theoretical views advanced in different parts of the work. But these are matters which only a careful exposition of the anatomy of similar structures in different classes of animals, together with experiments very accurately made and repeated, can decide.

We look forward to the completion of this work with great interest. It is one that was much wanted, and, notwithstanding some defects, is very valuable. In the execution of its plates we know of none with which it may be compared, excepting the already published work by Mr. Swan himself upon the nerves of the human body, a work which reflects honour not only on the author but on our country.

